# Co-administration of Systemic and Intralesional Zoledronic Acid in a Case of Fibrous Dysplasia: A Potentially Novel Therapy

**DOI:** 10.3389/fendo.2019.00803

**Published:** 2019-11-19

**Authors:** Sanjay Kumar Bhadada, Rimesh Pal, Ashwani Sood, Vandana Dhiman, Uttam Chand Saini

**Affiliations:** ^1^Department of Endocrinology, Post Graduate Institute of Medical Education and Research, Chandigarh, India; ^2^Department of Nuclear Medicine, Post Graduate Institute of Medical Education and Research, Chandigarh, India; ^3^Department of Orthopedics, Post Graduate Institute of Medical Education and Research, Chandigarh, India

**Keywords:** fibrous dysplasia (FD), bisphosphonate, zoledronate, local bisphosphonate, implant fixation

## Abstract

Fibrous dysplasia (FD) is a benign bone lesion characterized by replacement of normal bone with abnormal fibrous tissue, clinically manifesting as deformities, bone pains, and pathological fractures. The standard medical management for FD includes systemic bisphosphonate therapy. The efficacy of systemic bisphosphonate is however limited with minimal functional improvement and pain relief. Keeping the above lacunae in mind, we have made a solitary attempt at treating FD with locally administered zoledronic acid. A 25-year-old gentleman had presented to our institute with swelling and pain involving the left thigh and left lower leg. He was diagnosed as having polyostotic FD, confirmed on bone histopathology. He was administered 4 mg of zoledronic acid intravenously while 1 mg of the drug was injected locally into the femoral lesion under ultrasound and fluoroscopy guidance. There were no peri-procedural complications. At 6 months follow-up, there was marked improvement in pain scores at the left thigh, while that at the left leg remained unchanged. In addition, repeat bone scintigraphy showed a 20.8% and 25.3% reduction in anterior and posterior uptake values, respectively, at the left femur while that at the left tibia remained unaltered.

## Background

Fibrous dysplasia (FD) is a benign bone lesion characterized by replacement of normal bone by an excessive proliferation of cellular fibrous connective tissue intermixed with irregular trabecula (1). Long bones are most commonly affected. Three percent of all the cases occur in association with café-au-lait macules and/or hyper-functioning endocrinopathy (most common being precocious puberty), an entity referred to as McCune-Albright syndrome (MAS) (2, 3). FD has three clinical patterns, namely monostotic, polyostotic, and craniofacial forms (1). Clinically, patients with monostotic FD are usually asymptomatic and have a limited tendency to progress; presentation with pain, limp, or radiological evidence of microfracture predicts disease progression (4). Instead, polyostotic FD usually present with bone pains, fragility fractures, deformities, and facial asymmetries (2). MAS patients have the most extensive bone disease and regularly experience multiple fractures requiring recurrent surgical interventions (5). Diagnosis is usually based on plain radiographs that show an expansile radiolucent “ground-glass” lesion. Isotope bone scintigraphy delineates the entire extent of the disease. Histopathology shows irregular trabeculae of woven bones (giving a “Chinese letter” pattern) without an osteoblastic rim (6). The lesions are often lined by an unusually large number of osteoclasts (7). FD results from activating mutations in *GNAS1* gene (most common being R201H substitution) that codes for G_S_α protein. Constitutive activation of G_S_α leads to overproduction of cAMP in bone marrow stromal cells (BMSCs) causing osteoblast maturation arrest and unrestricted proliferation of unorganized masses of fibro-osseous tissues (8). cAMP increases IL-6 production by BMSCs that activates osteoclasts with consequent bone resorption and expansion of FD lesions. Rarely, malignant transformation of FD can occur with reported prevalence ranging from 0.4 to 4.0% (6).

Medical treatment of FD involves use of bisphosphonates, either administered orally or intravenously. Bisphosphonates inhibit osteoclast-mediated further bone resorption, preserve cortical bone mass and thereby reduce fracture risk (9). In addition, oral alendronate therapy and intravenously administered zoledronate/pamidronate have been shown to reduce bone turnover and partially suppress disease activity in polyostotic FD with no significant effect on pain or functional parameters (10, 11). Keeping these limitations of currently available treatment modalities in mind, we went ahead with the combination of systemic and intralesional administration of zoledronic acid in a patient with polyostotic FD, an endeavor that has hitherto never been undertaken. The concept of intralesional administration of this drug stemmed from prior studies wherein local application of bisphosphonates have been used as a means of counteracting secondary bone resorption following bone grafting and promoting early implant fixation (12–19). Bisphosphonates, either incorporated into implants or surface coated onto implants prevent bone resorption and actively promote bone regrowth into endoprosthesis porosities, thereby extending the durability of implants (18). In a double-blinded randomized control trial of 50 patients, application of 1 ml of ibandronate to the tibial bone surface led to improved prosthesis fixation following knee replacement (16).

## Case Presentation

**Figure 1:**
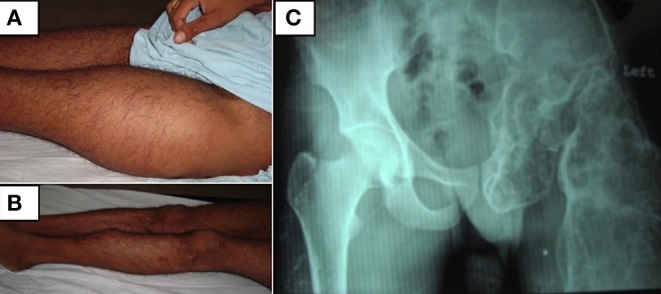
**(A,B)** Clinical photographs of the patient showing swellings involving the whole of left thigh and anterior part of the left mid-shin. **(C)** Radiograph of the pelvis showing a grossly deformed left femur and left ischium with multiple expansile lytic areas.

**Figure 2:**
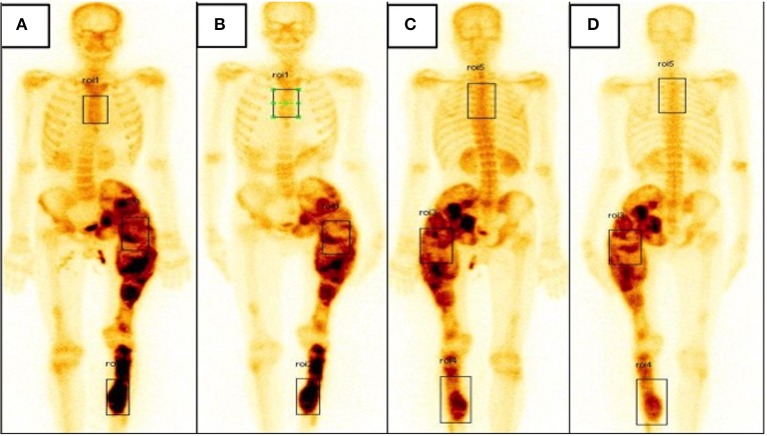
**(A,B)** Bone scintigraphy image, anterior view showing increased tracer uptake involving the left ischium, femur and part of tibia at baseline **(A)** and slightly reduced tracer uptake at the left femur when followed up at 6 months **(B)**. **(C,D)** Bone scintigraphy image, posterior view showing increased tracer uptake involving the left ischium, femur, and part of tibia at baseline **(C)** and slightly reduced tracer uptake at the left femur when followed up at 6 months **(D)**.

A 25-year-old gentleman presented to us with pain and swelling in the left thigh and shin. He had noticed the swelling at the age of 10 years and had been increasing ever since. Bony pain at the left thigh and shin was of recent onset. He denied any history of fractures or proximal muscle weakness. He was not on any medications other than over-the-counter analgesics for symptomatic pain relief. Physical examination revealed bony hard swellings involving the whole of left thigh and anterior part of the left mid-shin (Figures 1A,B). Pain was assessed using the subjective 11-point Numeric Pain Rating Scale that has been widely used for assessing severity of pain in FD (20, 21). The pain score at left thigh was 8/10 and at left leg was 7/10. There were no similar swellings in other parts of the body. He did not have any café-au-lait macules. Radiograph of the affected part showed a deformed left femur and left ischium with multiple expansile lytic areas (Figure 1C). Radiograph of the left leg showed a similar lesion involving the upper and mid-third of tibia. Bone scintigraphy showed increased tracer uptake involving the left ischium, femur and part of tibia (Figures 2A,C, anterior and posterior views, respectively). There was no evidence of increased tracer uptake in other parts of the skeleton. Bone biopsy from the femoral lesion showed irregular trabeculae of woven bones suggestive of FD. Biochemical panel revealed normocalcemia and normophosphatemia. He was vitamin D sufficient. Bone turnover markers [P1NP = 143 ng/ml (age-specific range: 38.5–86 ng/ml) and CTX = 920 pg/ml (age-specific range: 112–584 pg/ml)] were elevated. His thyroid function test, insulin-like growth factor 1 (IGF-1) and testosterone levels were all normal, ruling out any endocrinopathy and MAS.

After taking informed consent from the patient, 4 mg of zoledronic acid was administered intravenously. Simultaneously, 3 ml of zoledronic acid, containing 1 mg of the drug was injected within the dysplastic lesion involving the left femur. Under ultrasound guidance, a Jamshidi needle was inserted into the shaft of the left femur along the lateral aspect at a junction of the upper and middle third. The position of the tip of the needle was confirmed using C-arm fluoroscopy. The trocar was removed and subsequently the required amount of the drug was slowly injected. There were no intra-procedural complications. The following day he developed high-grade fever that lasted for 2 days, likely attributable to systemically administered zoledronate.

At 6 months follow-up, there was no noticeable change in the swelling of the left thigh or left shin. However, pain scores at left thigh was reduced to 4/10 while that in left shin remained unchanged at 7/10. He had not developed any fractures in this interim period. Complete biochemical panel was unremarkable. Bone turnover markers were slightly reduced from baseline values (P1NP = 105.3 ng/ml, CTX = 710 pg/ml). Bone scintigraphy was repeated; when compared with the baseline scan, there was a reduction in uptake at left thigh and no change at left shin. The anterior uptake values at left thigh and left tibia at baseline were 3.07 and 3.55, respectively (Figure 2A); at 6 months, the corresponding values were 2.43 and 3.60, respectively (Figure 2B). Similarly, the posterior uptake values at left thigh and left shin at baseline were 2.25 and 1.46, respectively (Figure 2C); the corresponding values at 6 months follow-up were 1.68 and 1.48, respectively (Figure 2D) (summarized in Table 1).

**Table 1:** Table showing anterior and posterior uptake values on bone scintigraphy at the left thigh and left tibia pre and 6 months post intravenous and intralesional zoledronate therapy.

	**Left thigh**		**Left shin**	
	**Anterior uptake value**	**Posterior uptake value**	**Anterior uptake value**	**Posterior uptake value**
Pre-zoledronate	3.07	2.25	3.55	1.46
Post-zoledronate	2.43	1.68	3.60	1.48
Percentage change (%)	−20.8	−25.3	+1.4	+1.3

## Discussion

We have demonstrated efficacy of intralesional zoledronate over and above systemic bisphosphonate in the treatment of FD. Our index patient was administered systemic zoledronate at a recommended dose of 4 mg followed by intralesional bisphosphonate at the femoral lesion with the tibial lesion acting as an auto-control. At 6 months follow-up, there was marked improvement in the pain scores at left thigh with no change at left shin. Moreover, bone scintigraphy showed reduction in uptake at left femur and no change at left tibia, adding testimony to the fact that locally administered bisphosphonate is effective and perhaps acts synergistically with the systemically administered drug in markedly reducing osteoclast activity.

Management of FD is challenging with systemic bisphosphonates being the treatment of choice. Systemic bisphosphonates do reduce bone turnover markers, however, improvement in pain and functional parameters are debatable (10, 11). There is a dire need for new treatment modalities for the management of FD. Keeping this in mind, we came up with the innovative idea of intralesional injection of bisphosphonate in addition to the conventional administration of the drug by intravenous route. The idea stemmed from observations that locally administered bisphosphonates is an effective means of enhancing bone-implant fixation. Bisphosphonates, acting locally has been shown to suppress osteoclast function at the bone-implant interface; at the same time it has been shown to activate osteoblast activity, promoting bone-implant integration (18). Systemic bisphosphonates administered in a patient with FD are concentrated at the sites of the lesions, however, only 50% of the intravenously administered drug is available for incorporation in the bone matrix (22), hence, the concentration can be expected to be much lower compared to the locally injected drug. At such low concentrations, local bone turnover at the FD lesions are minimally suppressed, as was evident at the left tibia of our index patient. When administered locally over and above the systemically administered one, the two perhaps acts synergistically leading to more profound suppression of local bone turnover, resulting in reduction of uptake values and pain scores. In addition, the osteo-anabolic activity of local bisphosphonate coupled with increased osteoclast inhibition might have contributed to transformation of immature woven to more mature lamellar bone and subsequently reduced tracer uptake on scintigraphy. A repeat bone biopsy at follow-up would have been required to prove or disprove the aforementioned hypothesis, however, the patient did not consent for the same. Moreover the higher concentrations of the drug achieved locally with intralesional administration might allow for smaller and less frequent dosing. The efficacy of locally administered bisphosphonate can further be augmented by the use of drug-coated scaffolds or carriers that would increase the biological permanence of the drug at the desired site of action (23). In addition, this would further reduce the systemic side effects of bisphosphonate (that includes infusion-related reactions, myalgias, cutaneous reactions, osteonecrosis of jaw, atrial fibrillation, uveitis, and nephrotoxicity) which is otherwise negligible with local application of the drug (24, 25).

In conclusion, we have demonstrated a novel approach to effectively treat fibrous dysplasia. How practical will be this treatment modality in polyostotic FD is certainly debatable, however, it can certainly be considered as an option in patients with monostotic FD. A phase 3 trial on the efficacy of local bisphosphonate in reducing recurrence rates in extremity giant cell tumor of bone is currently underway (NCT 03295981). Similar, large-scale randomized-controlled trials need to be undertaken comparing the efficacy of intralesional bisphosphonate over and above the systemic drug in FD.

## Data Availability Statement

All datasets for this study are included in the article/supplementary material.

## Ethics Statement

The studies involving human participants were reviewed and approved by Institute Ethics Committee PGIMER, Chandigarh. The patients/participants provided their written informed consent to participate in this study. Written informed consent was obtained from the individual(s) for the publication of any potentially identifiable images or data included in this article.

## Author Contributions

SB had conceived the idea of intralesional zoledronate therapy. RP had prepared the manuscript. AS had provided the bone scintigraphy images. VD had edited and revised the manuscript. US had performed bone biopsy and helped us in administering zoledronate intralesionally. All the four authors had approved the final revised version of the manuscript.

### Conflict of Interest

The authors declare that the research was conducted in the absence of any commercial or financial relationships that could be construed as a potential conflict of interest.
